# A ^1^H-NMR Based Study on Hemolymph Metabolomics in Eri Silkworm after Oral Administration of 1-Deoxynojirimycin

**DOI:** 10.1371/journal.pone.0131696

**Published:** 2015-07-06

**Authors:** Ming-Jie Deng, Xiao-Dong Lin, Qiu-Ting Lin, De-Fu Wen, Mei-Ling Zhang, Xian-Qin Wang, Hong-Chang Gao, Jia-Ping Xu

**Affiliations:** 1 School of Life Sciences, Anhui Agricultural University, Hefei, China; 2 Analytical and Testing Center of Wenzhou Medical University, Wenzhou, China; Institute of Plant Physiology and Ecology, CHINA

## Abstract

We aimed to investigate whether 1-deoxynojirimycin (DNJ) modulates glycometabolism and has toxicity in Eri silkworm (*Samia cynthia ricini*, Saturniidae). In this paper, hemolymph metabolites were used to explore metabolic changes after oral administration of DNJ or mulberry latex and to characterize the biological function of DNJ at the metabolic and systemic levels. Hemolymph samples were collected from fourth-instar larvae of Eri silkworm and ex-vivo high-resolution ^1^H nuclear magnetic resonance (NMR) spectra were acquired from the collected hemolymph samples. Then the obtained spectra were analyzed by principal component analysis (PCA) and independent-samples t-test. Metabolic pattern recognition analysis of hemolymph samples indicated that the groups of 0.25% DNJ, latex, and the mixture of 0.5% DNJ and latex (1:1) were significantly different from the control group. Moreover, compared to the control group, the groups of 0.25% DNJ, latex, and the mixture of 0.5% DNJ and latex (1:1) showed the decreased levels of citrate, succinate, fumarate, malate, and glutamine in hemolymph, the groups of 0.25% DNJ and the mixture of 0.5% DNJ and latex (1:1) showed the increased levels of trehalose and lactate. In addition, mulberry leaves exude latex was highly toxic to Eri silkworm because rich unidentified high-molecular-weight factor (s) acted as toxic substances. In our results, latex caused 20 deaths among 50 fourth-instar larvae of Eri silkmoth, but DNJ or the mixture did not caused death. All these results suggest that DNJ has a positive impact on the reverse glycometabolism by modulating glycometabolism and inhibiting glucogenesis and energy metabolism. DNJ is a secure substance as a single-ingredient antidiabetic medicine due to its nontoxicity and bioactivity.

## Introduction

**Fig 1:**
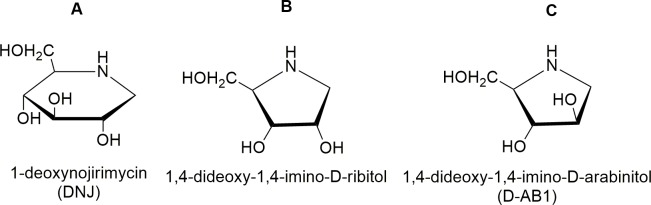
Molecular structures of 1-deoxynojirimycin (A), 1,4-dideoxy-1,4-imino-D-ribitol (B), and D-AB1 (C).

Mulberry leaves have been widely cultivated for rearing the silkworm *Bombyx mori* (*B*. *mori*) in ancient times. Domesticated silkworms can grow adaptively on mulberry leaves, so the defense response and toxic properties of the leaves to the insect are generally ignored [1,2]. In the defense response against silkworms, mulberry leaves exude latex containing rich 1-deoxynojirimycin (DNJ), 1,4-dideoxy-1,4-imino-D-ribitol, and 1,4-dideoxy-1,4-imino-D-arabinitol (D-AB1) (Fig 1A–1C). All the above three substances are glycosidase inhibitors. These iminosugar inhibitors are lethal to Eri silkworms, a generalist herbivorous insect. However, they are safe to domesticated silkworms on mulberry leaves, *B*. *mori* [3]. Mulberry leaves will lose their toxicity to *Samia cynthia ricini* (*S*. *cynthia ricini*) when latex is eliminated from mulberry leaves through cutting leaves into slim pieces and subsequent rinsing, interpreting that the defensive ability of mulberry leaves is totally dependent upon toxic latex. In our study, after Eri silkworms were fed with 5 μL of latex, among 50 larvae, 20 larvae died and the growth of other 30 larvae was significantly retarded with frequent vomiturition. In the meanwhile, when Eri silkworms were fed with 5 μL of 0.25% DNJ or the mixture of latex and 0.5% DNJ, no larva died (n = 50 in each group) and the morphology of larvae could not be distinguished from the controls by visual examination. DNJ is a characteristic constituent of mulberry latex and DNJ concentrations in mulberry latex are different between cultivated varieties and natural populations [4]. Among the above three iminosugar inhibitors, only DNJ, was found in the latex from *Morus alba* (*M*. *alba*, a Chinese local species) [1].

DNJ is a natural D-glucose analogue with promising physiological activity *in vivo*. It can inhibit α-glucosidase in the small intestine and the liver, enhance the expression of adiponectin mRNA in whiter adipose tissue, delay the senescence of blood vessels and affect the reverse cholesterol metabolism [5–7]. Due to the potential medicinal value of DNJ, DNJ-enriched products may represent a therapy or oral treatment of diabetes mellitus, and numerous DNJ tablets have been commercially available in many countries [7]. These studies illustrate the bioavailability of DNJ, but their DNJ content has not been specified. Previous studies focused on the antidiabetic role of DNJ in rats or other mammals, but direct physiological evidences clearly elaborating the detailed physiological mechanisms of glycometabolism or toxicological effects of DNJ against insects were not reported. Glucogenesis, glycolysis, and tricarboxylic acid (TCA) cycle related to glycometabolism exist in all aerobic organisms and the hypoglycemic characteristic of DNJ in mammals is similar to that in non-specialist herbivorous insects [7–9]. Furthermore, some conservative proteins, such as α-glucosidase, glucokinase, pyruvate kinase, and glucose-6-phosphatase, are involved in DNJ metabolism in mammals and insects [10,11]. This suggests that physiological mechanisms of glycometabolism induced by DNJ in mammals may be an inheritable characteristic from generalist herbivorous insect. Our study focused on the changes of intermediates in basic metabolic pathways in hemolymph of Eri silkworms caused by feeding DNJ. We found that metabolic disorders were related to the reversed regulation of glycometabolism by DNJ. This result indicated the therapeutic application value of DNJ in human beings.

In metabonomics, dynamic metabolic responses of living organisms to genetic modifications or pathophysiological stimuli were quantified [12,13]. Metabonomic techniques can harmlessly and dynamically detect the changes in metabolites in biological fluids, evaluate the toxic effect of the tested substances, provide the information related to drug toxicity, and evaluate the clinical effects of drugs on gene expression by integrating its temporal and spatial effects [14]. In the NMR-based metabonomics, ^1^H nuclear magnetic resonance (^1^H NMR) spectra of samples are combined with statistical analysis. The NMR-based metabolomics technique is considered as a valuable analysis technique of biological samples because it can be used to fast detect the alterations in systematic metabolite profiles [14]. In recent years, NMR-based metabolomics have been widely used for assessment of disease mechanisms, prediction of human diseases and early diagnosis of many organ-based metabolite markers associated with various cancers [15–17]. Given its usefulness in evaluating systemic responses to subtle metabolic perturbation, metabolomics can be used for the biological evaluation of DNJ as well as the identification of its potential medicinal properties [18–20].

The paper aims to address two questions. The first one is “whether DNJ is toxic to Eri silkworm, *S*. *cynthia ricini*, just like mulberry latex is toxic to generalist insects?” The second is “how do DNJ and latex modulate glycometabolism in Eri silkworm, *S*. *cynthia ricini*?” The replies to the above two questions will be conducive to the verification of the safety aspect of DNJ and the understanding of effect of DNJ on insects and plant–herbivore interaction.

## Materials and Methods

### Insects

Eri silkworms were maintained in the laboratory to identify the effects of mulberry latex and DNJ on nonmulberry-specialist caterpillars. Mulberry trees are not the natural host plant of *S*. *cynthia ricini* larvae. *S*. *cynthia ricini* larvae have been applied to detect and assess the levels of plant defense responses to herbivorous insects [1,3]. In the experiment, we adopted newly molted fourth-instar larvae *S*. *cynthia ricini* (obtained from The Sericultural Research Institute of Chinese Academy of Agricultural Sciences, Zhenjiang, and then maintained in our laboratory of Wenzhou Medical University) to evaluate the effects of mulberry latex and DNJ on the herbivorous insects. Hatched larvae were reared on castor oil leaves ad libitum under controlled temperature (20–25°C) and humidity until the end of the experiments.

### Mulberry latex

Mulberry latex was gathered from the wild plants of *Morus alba* in the cultivated garden of Wenzhou Medical University, Wenzhou, China (28°N, 120°E) by directly cutting the petioles. The administration procedures of mulberry had been approved by the Institutional Plant Committee and Use Committee of Wenzhou Medical University (Document No.: wydw2012-0083). The latex from this population contained only 0.32±0% of DNJ, whlie 1,4-dideoxy-1,4-imino-D-ribitol or D-AB1 was not detected in the latex [21]. The latex from the cut petioles was gathered with test tubes, maintained at 4°C, and then used in 12 h.

### Experimental design and sample collection

Firstly, 150 newly molted fourth-instar larvae were randomly selected, and divided into three groups, which were then respectively fed with 0.25% DNJ (J&K Chemicals), latex, and the mixture of 0.5% DNJ and latex (1:1) at a single dosage of 5 μL. The three groups were respectively named the DNJ group, latex group, and mixture group, respectively. The other 50 larvae were fed with 5 μL of ultrapure water (Millipore, America) as the control group. After continuous administration for 2 days, about 50 μL of hemolymph was collected by cutting the feet of the individual from each group on Day 3 and hemolymph from 5 individuals was brought together to form one sample. The collected hemolymph was added with a small amount of thiourea and immediately kept at -80°C.

### Preparation of hemolymph samples and acquisition of ^1^H NMR spectra

Hemolymph samples were firstly thawed. Then 100 mL of the supernatant was diluted with 60 μL of D_2_O for a field-frequency lock and 400 mL of phosphate buffer (0.2 mM Na_2_HPO_4_/NaH_2_PO_4_, pH 7.4) for minimizing pH variation before NMR analysis. At 4°C, the mixed hemolymph was centrifuged at 12,000×g for 10 min and then 500 μL of supernatant was pipetted into 5-mm NMR tubes. In the experiment, Bruker AVANCE III 600 MHz NMR spectrometer equipped with a triple resonance probe and a z-axis pulsed field gradient was adopted to acquire NMR spectra at 300 K. In order to suppress the NMR signals from tardily tumbling molecules including lipids and proteins and maintain those signals from some lipid components and low-molecular-weight compounds, the Carr–Purcell–Meiboom–Gill (CPMG) pulse sequence with the fixed relaxation delay, 2nτ of 120 ms, was adopted [22]. Typically, 256 scans were acquired into 64 K data points (the spectral width of 12,336 Hz, a relaxation delay of 4 s and the acquisition time of 2.66 s). Before Fourier transformation, the spectra were weighted according to a Gaussian function with an exponential line-broadening of 0.3 Hz for the free induction decay. All the phase and baseline of the obtained spectra were corrected manually according to chemical shifts of methyl peak of lactate (CH_3_, δ 1.33) [23].

### Data refinement and multivariate pattern recognition analysis

After the spectra were corrected, with the Topspin 2.1 software package, each spectrum was segmented into different chemical shift regions with the same width of 0.01 ppm, which was equivalent to the region of δ of 9.5~0.5, for multivariate pattern recognition analysis. In the analysis, the spectra region corresponding to residual peak from water resonance (5.0–4.6 ppm) was removed to zero. The data of remaining spectral segments were exported to Microsoft Excel. Before multivariate analysis, the peaks should be normalized to the sum of spectra. Then, the concentrations of the metabolites were expressed as relative peak areas. The metabolite data derived from the control and treatment groups were imported into SIMCA-P 12.0 software (Umetrics, Umea, Sweden) to perform principal component analysis (PCA). In order to differentiate the metabolic profiles obtained with hemolymph samples of the four groups, an unsupervised PR method, PCA, was adopted to process the data obtained from the hemolymph samples. Based on PCA, the metabolites which could be used to differentiate the control group from each treatment group were identified and integrated. From the integrated data, the relative intensity of each metabolite was then calculated [15]. Each point indicated an individual spectrum of a sample and could be differentiated from other points with the first two principal components, PC1 and PC2. Thus, the data could be displayed via plotting with the scores of PC1 and PC2. In the plots, each point indicated a single NMR spectral region segment and the metabolites related to differentiating the groups were exhibited with corresponding loading plots [19]. A coefficient of variation plots showed the differences in the metabolites among the groups, which allowed the interpretation because the loadings resembled NMR spectra. The loading plots and score plots could complement each other. The goodness of fit and model validity were tested and computed with the parameters of R^2^ and Q^2^, where R^2^ represented the sum of the square of the entire X and Q^2^ was the fraction of cross-validation-explained variation with the increase of the reliability [24].

### Statistical analysis

In order to obtain significant differences among metabolic changes, we analyzed the normalized integral values with SPSS 13.0 software. In the statistical analysis, independent-samples t-test was used to analyze the acquired data. If the *P*-value was calculated to be lower than 0.05, the difference was believed to be statistically significant. Data were expressed as mean±standard deviations (SD).

## Results

### Toxicity of DNJ and latex on Eri silkworm, *S*. *cynthia ricini*

To investigate the defense role of DNJ in mulberry latex, the effects of DNJ and latex on Eri silkworms were studied. The latex from *Morus alba* contained 0.32±0% of DNJ, while1,4-dideoxy-1,4-imino-D-ribitol or D-AB1 were not detected [21]. Among different groups, the concentration of DNJ was gradually increased in the sequence: 0.25% DNJ, latex and the mixture of latex and 0.5% DNJ. After Eri silkworms were fed with 5 μL of latex containing 0.32% DNJ, among 50 larvae, 20 larvae died and the growth of other 30 larvae was significantly retarded with frequent vomiturition. However, after Eri silkworms were fed with 5 μL of 0.25% DNJ or the mixture of latex and 0.5% DNJ, no larva died (n = 50 in each group). These results (Table 1) indicated that unidentified high-molecular-weight (UHMW) factor (s) in latex might be responsible for its toxicity. The toxicity of latex depended on the concentration of these UHMW factor(s). DNJ is a secure substance due to its nontoxicity.

**Table 1:** Concentrations of DNJ in four groups and their mortality rate, respectively.

Groups	Concentrations of DNJ(%)	Mortality rate(%)
H_2_O	0	0
0.25% DNJ	0.25	0
Latex	0.32	40
Mixture	0.41	0

^1^H NMR spectral analysis of hemolymph samples

**Fig 2:**
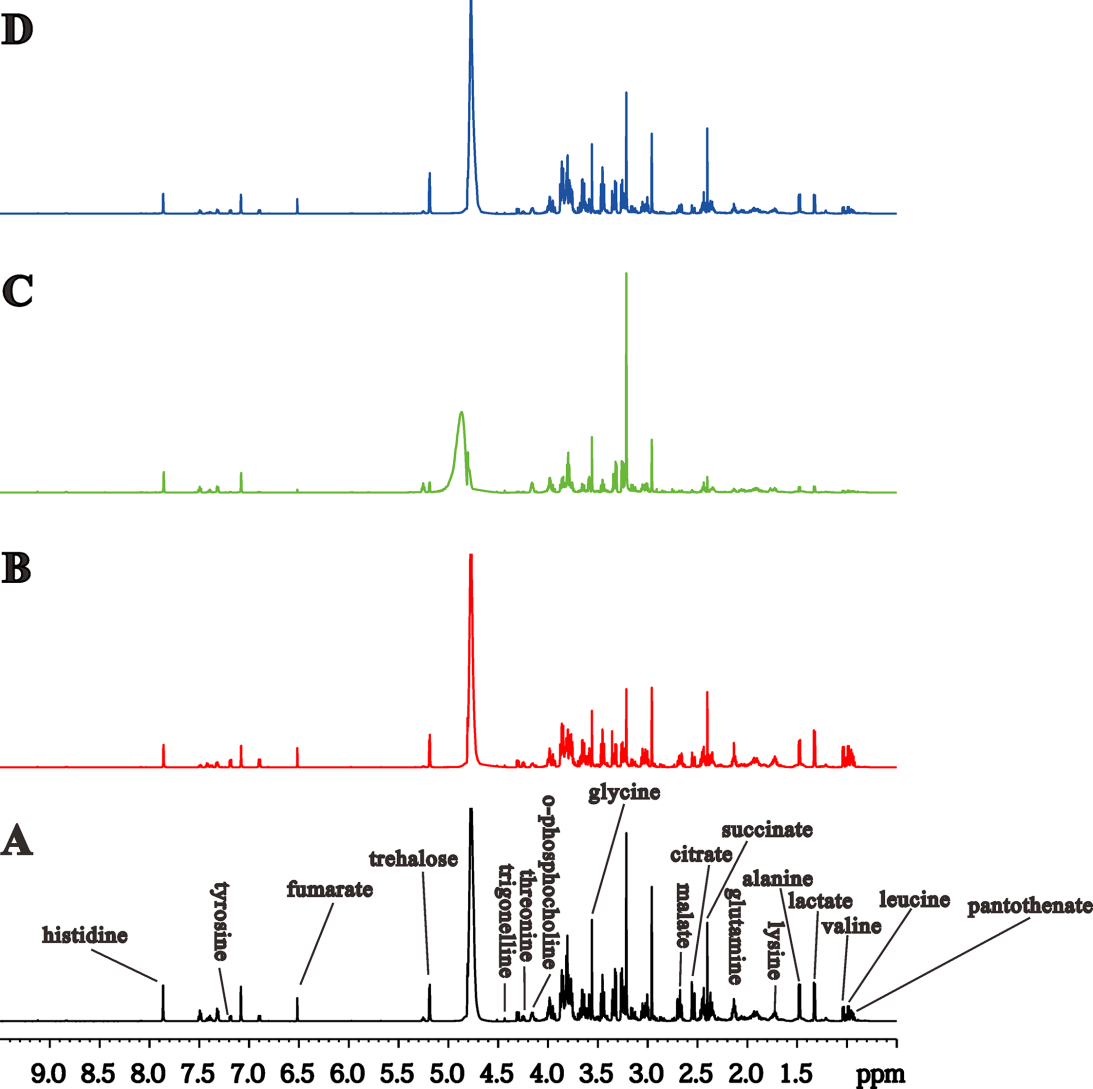
Typical ^1^H NMR spectra of hemolymph of Eri silkworms of four groups: control group (A), 0.25% DNJ group (B), latex group (C), and the mixture group (D).

In the typical ^1^H-NMR spectra of hemolymph samples from Eri silkworms in four groups (Fig 2A–2D), the spectrographic resonances of the metabolites were designated based on our published work [19,24–26] as well as the 600-MHz library in Chenomx NMR Suite 7.0 (Chenomx Inc., Edmonton, Canada). In addition, partial samples were checked to identify the assignments based on 1D ^1^H NMR spectra according to 2D ^1^H-^1^H COSY spectra obtained under solvent suppression. Based on the ^1^H-NMR spectra of the hemolymph, we simultaneously detected several endogenous metabolites, including leucine (δ0.95), valine (δ1.04), lactate (δ1.32), alanine (δ1.48), lysine (δ1.72), glutamine (δ2.13), succinate (δ2.40), citrate (δ2.54), malate (δ2.66), glycine (δ3.56), o-phosphocholine (δ4.16), threonine (δ4.25), trigonelline (δ4.44), trehalose (δ5.19), fumarate (δ6.52), tyrosine (δ7.19), and histidine (δ7.86), (S1 Table).

### Pattern recognition analysis of hemolymph samples

**Fig 3:**
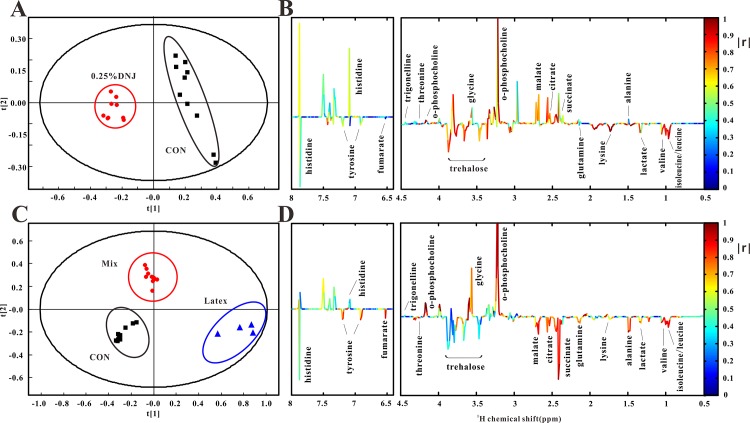
Results of principal component analysis (PCA) of ^1^H NMR spectra of hemolymph samples. PCA score plots (A) obtained with ^**1**^H NMR spectra of the 0.25% DNJ group and control group (R^**2**^ = 0.712, Q^**2**^ = 0.64, ■control group, ●0.25% DNJ group) and coefficient-coded loading plots (B). PCA score plots (C) obtained with ^**1**^H NMR spectra of the 0.25% DNJ group, mixture group, and control group (R^**2**^ = 0.858, Q^**2**^ = 0.636, ■control group, ●mixture group, ▲latex group) and coefficient-coded loading plots (D).

In order to elaborate DNJ-induced changes in the metabolism system and determine related metabolic pathways, NMR spectra of hemolymph were segmented to perform principal component analysis (PCA, Fig 3). As shown in Fig 3A, the 0.25% DNJ group could be obviously discriminated from the control group along the PC1 direction (R^2^ = 0.712, Q^2^ = 0.64), indicating that the cluster of the 0.25% DNJ group had diverse characteristics compared to the control group. Fig 3B shows corresponding loading plots obtained with color-coded correlation coefficients (|r|) between the 0.25% DNJ group and the control group and display the variables accounting for the differentiation among different groups. The positive regions in the loading plot corresponded to the decreased metabolites in the hemolymph of the 0.25% DNJ group, whereas negative regions corresponded to the increased metabolites in the hemolymph of the 0.25% DNJ group. Thus, Fig 3B revealed that the Eri silkworms of the 0.25% DNJ group excreted the higher levels of leucine, valine, lactate, lysine, and trehalose, but the lower levels of succinate, citrate, o-phosphocholine, glycine and trigonelline than those in the control group.

**Fig 4:**
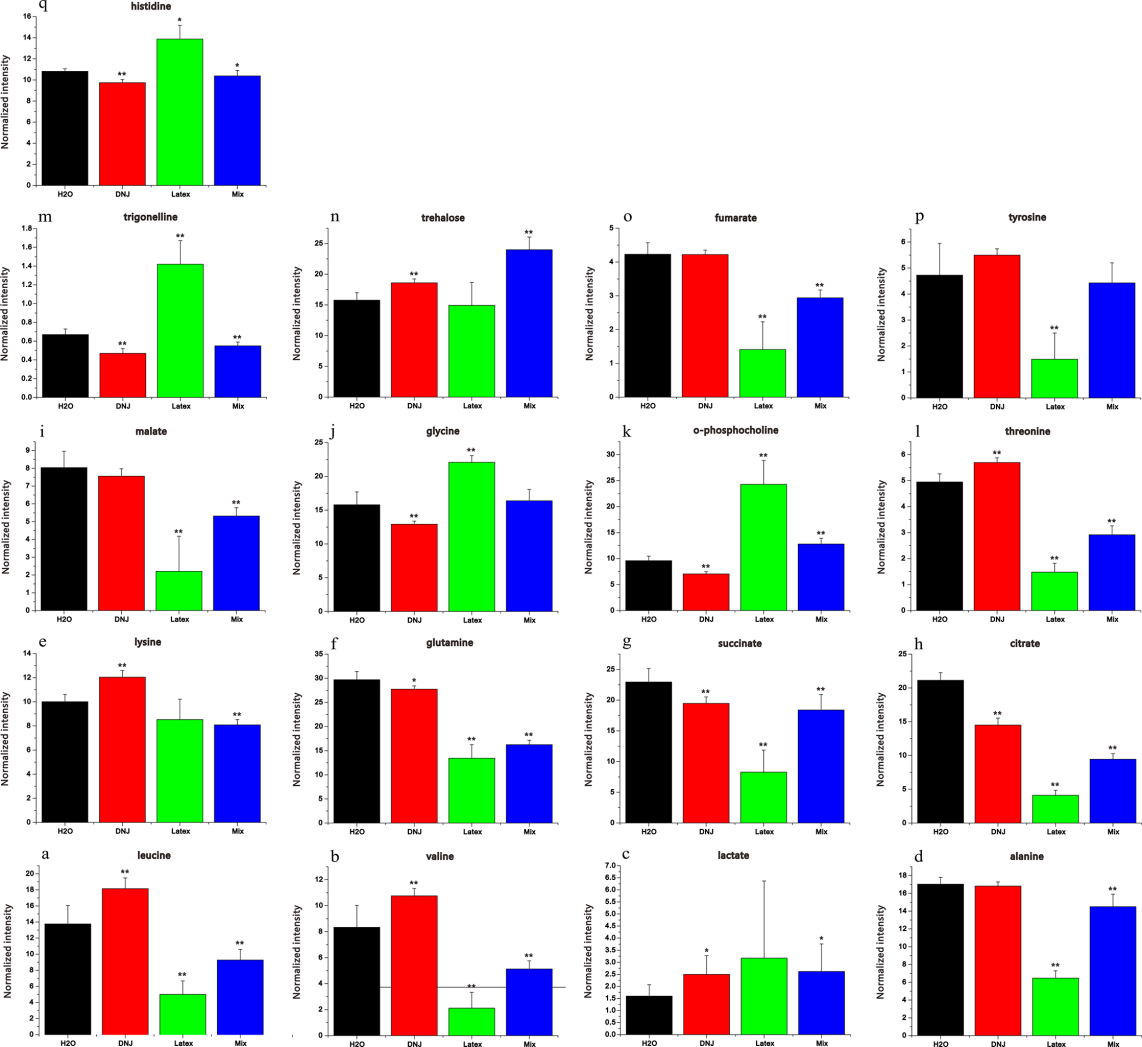
Metabolic changes found in ^1^H NMR spectra of hemolymph samples of the control group, 0.25% DNJ group, latex group, and the mixture group, respectively. Values are expressed as mean±SD. ^*^ and ^**^ mean the significant (*P*<0.05) and extremely significant differences (*P*<0.01) compared to the control group.

According to the PCA score plots obtained from the hemolymph of latex, mixture and the control groups, three groups showed significant differences along the PC1 direction and PC2 direction (Fig 3C, R^2^ = 0.858, Q^2^ = 0.636). The significant differences between the control group and two treatment groups (latex group and the mixture group) indicate that metabolic turbulence occurs in Eri silkworms of the latex and mixture groups. The corresponding loading plot (Fig 3D) shows that the variables related to succinate, o-phosphocholine, glycine, trehalose and other metabolites are mainly responsible for the differentiation (Fig 4).

### Quantitative analysis of metabolites

S1 Table shows corresponding integral levels of metabolites in hemolymph extracts from Eri silkworms, *S*. *cynthia ricini*, of the four groups. The variation tendency of the metabolites obtained by quantitative analyses of statistics is consistent with those indicated by the PCA loading plots (Fig 3). The results suggested that trehalose increased significantly in hemolymph of Eri silkworms fed with 0.25% DNJ or the mixture. The increase indicated the inhibition on trehalase and the hydrolysis of trehalose, which is the dominant blood sugar in an insect (Fig 4N). One additional glycolysis-related product, lactate, was increased in hemolymph after feeding with 0.25% DNJ or the mixture (Fig 4C). As significant tricarboxylic acid (TCA) cycle intermediates, fumarate, citrate, succinate, and malate in hemolymph in three treatment groups were decreased in various degrees (Fig 4G–4I and 4O). As the substrate of gluconeogenesis, glutamine was decreased in hemolymph after feeding with 0.25% DNJ, latex or the mixture and reached the minimum in the latex group (Fig 4F). Excretions of leucine, valine, lysine and threonine were enhanced in the 0.25% DNJ group and decreased in the latex and mixture groups (Fig 4A–4B, 4E and 4L). In addition, compared to the control, the 0.25% DNJ group showed the significantly decreased levels of glycine, o-phosphocholine, trigonelline, and histidine (Fig 4J–4K, 4M and 4Q). The level of alanine increased in the latex and mixture groups (Fig 4D). The level of tyrosine was decreased in the latex group and showed no significant difference with that in other groups (Fig 4P).

## Discussion

**Fig 5:**
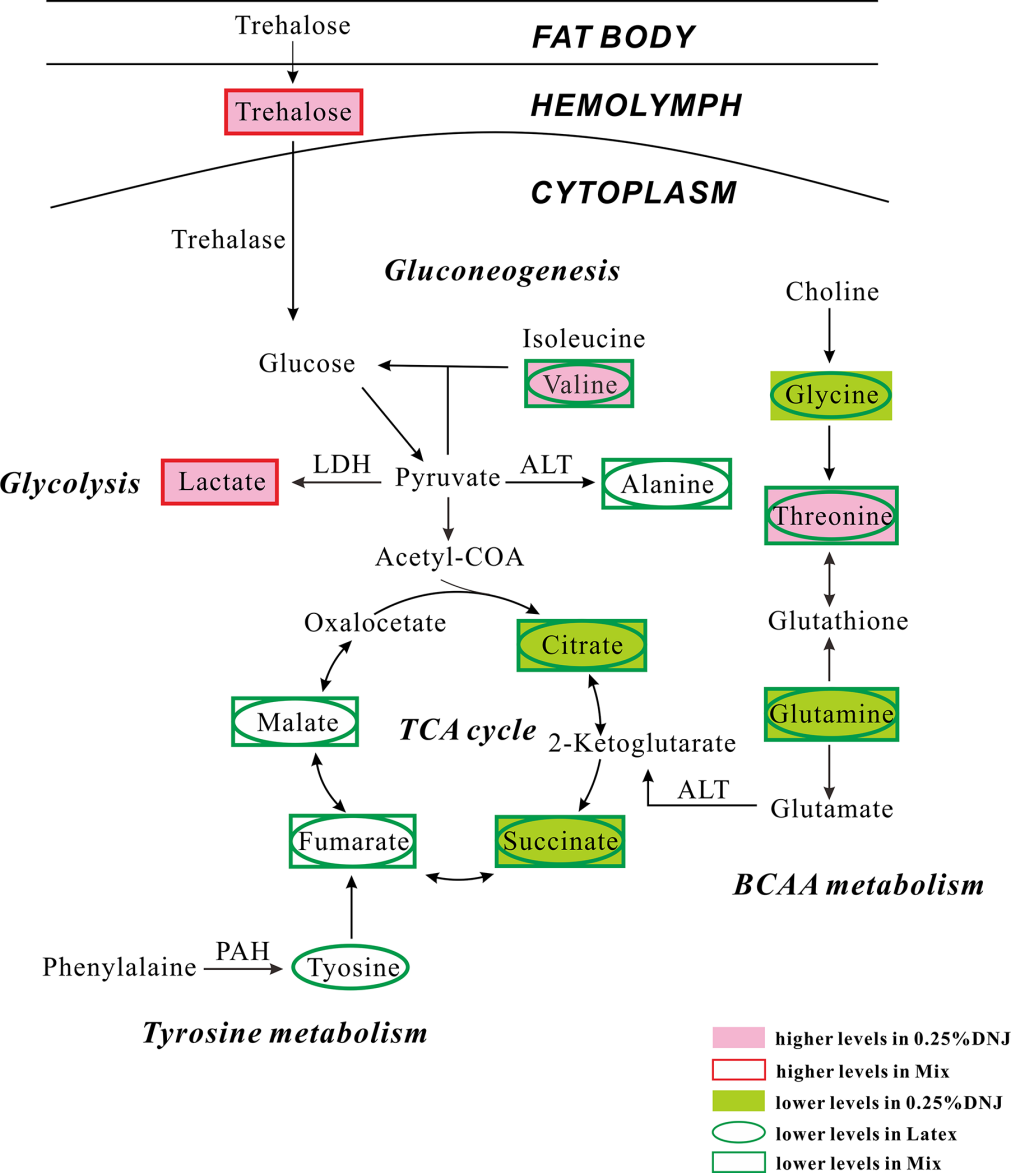
Schematic diagram of the metabolic pathways. The metabolite changes detected by ^**1**^H NMR hemolymph analysis and the pathway referenced to the KEGG database show the interrelationship of the identified metabolic pathways involved in the experimental Eri silkworms. Metabolites with increased and decreased levels compared to control silkworms are indicated in red and blue.

As an important class of glycosidase suppressants, iminosugars recently became the study focus of potential therapeutic agents [27–29]. As one kind of iminosugars, DNJ shows the high in-vitro inhibition activity on α-glucosidase. Its in-vivo efficacy and mechanism are obscure, especially in caterpillars. In our study, the ^1^H NMR spectra of hemolymph samples of Eri silkworms after different preconditioning treatments revealed the significant changes in the concentrations of critical endogenous metabolites compared to those in the control group. The global metabolic abnormalities related to specific metabolic pathways, such as glycolysis, gluconeogenesis, TCA cycle, and energy and amino acid metabolism, were determined. The results expedite us to explore the metabolism mechanisms of DNJ. Fig 5 illustrates the altered metabolic pathways in hemolymph of Eri silkworms after oral administration of 0.25% DNJ, latex and mixture based on the KEGG database (http://www.genome.jp/kegg/pathway.html).

### Glycometabolism

Carbohydrate metabolism is essential for the physiological balance of living organisms. The regulation of serum glucose levels by insulin and glucagon in mammals is parallel to the regulation of hemolymph carbohydrate availability in insects. In Eri silkworms and other lepidopterans, incorporated fatty acids are converted and stored as diacylglyceride in lipophorin, amino acids in storage proteins, and sugars in trehalose, mostly in the hemolymph [30,31]. Hemolymph trehalose concentration is up-regulated by a hypertrehalosemic hormone (HTH), which has been isolated from corpora cardiac (CC) of various orders of insect, and down-regulated by a hypotrehalosemic factor in brain extracts in *Calliphora erythrocephala* and *Phormia regina* [32,33]. Trehalose is a disaccharide comprising two glycosidically linked glucose units found in all the insects [34]. In insects, trehalose is the main metabolic source of energy required for flight and very important in the physiological adaptation to the environment [34,35]. In our study, the concentration increase of trehalose related to the glycometabolism was observed in hemolymph samples of Eri silkworms of the 0.25% DNJ group and the mixture group (Fig 4N). The results indicated that the utilizable pathways of trehalose were inhibited by the potency of DNJ. It is known that the trehalose concentration in the hemolymph is regulated homeostatically in the short term (several minutes and hours) and influences insect development in the long term (days and months) [30,34,36,37]. Meanwhile, as the main source of glucose, trehalose may be the key intermediate regulatory product and the disaccharide can be rapidly utilized as an energy source when insects are hungry [34,38]. We thereby hypothesize that the increased levels of trehalose in the hemolymph may be an important event in the physiological role of DNJ, which results in the impaired hydrolytic pathway of trehalose.

In the paper, the increased concentration of lactate related to glycolysis was observed in hemolymph samples of the 0.25% DNJ and mixture groups (Fig 4C). As one additional glycolysis-related product, the augmented lactate levels suggested the enhancement of glycolysis and the decrease of glucose level. This result was also consistent with previous results that DNJ inhibited intestinal glucose absorption and increased the activity of glucose glycolysis enzymes via the relative enhancement in protein expression [10,39]. Moreover, lactate can be used as the energetic substrate in the glycolytic process [40]. The significant increase of lactate in hemolymph in our study might be the consequence of the organism switching to the accumulation of glycolysis products and the reduction of energy homeostasis in Eri silkworms. In addition, alanine can be transformed into glucose (Fig 4D) and the transformation process is called the gluconeogenesis [41]. As a consequence, alanine may be considered as the precursor of glycogenic amino acid. The decreased concentration of alanine in hemolymph observed in the latex group and the mixture group suggested the enhanced gluconeogenesis in these groups, but the decreased alanine level was not observed in the 0.25% DNJ group.

### Tricarboxylic acid (TCA) cycle and energy metabolism

The TCA cycle, also known as the citric acid cycle, is a succession of biochemical reactions in all aerobic organisms to generate energy through the oxidation of cetyl-CoA derived from proteins, fats, and carbohydrates into carbon dioxide and adenosine triphosphate (ATP) [36,37]. In the meantime, the cycle supplies precursors of certain amino acids as the substances in other biochemical reactions and usable chemical energy for cellular activity [38–41]. Its central role in many biological pathways suggests that it is the hinge of carbohydrate, fat, and protein metabolism. In hemolymph samples of Eri silkworms, a series of metabolites involved in TCA cycle are perturbed as a result of feeding with 0.25% DNJ, latex, or the mixture. As important TCA cycle intermediates, the levels of citrate and succinate decreased in hemolymph of all experimental groups compared to that in the control group (Fig 4J and 4H). The levels of malate and fumarate in hemolymph were decreased in the latex and mixture groups (Fig 4I and 4O). The reductive metabolite levels indicated the significant metabolic changes in the TCA cycle in Eri silkworms of all the treatment groups. For further elaboration, 0.25%DNJ inhibited the TCA cycle via partial intermediate products involved in the cycle, such as citrate and succinate. The decrease in the relative concentrations of most intermediates of TCA cycle might be caused by the systemic stress generated under the synergistic effects between DNJ and the UHMW factor(s) in latex. It is unclear whether pure DNJ influences other intermediates involved in TCA cycle at the higher concentration in our work.

Trehalose is the primary sugar and the main energy source in hemolymph of insects and can protect them from extreme environmental conditions [35]. From above-mentioned results, the enhanced trehalose levels in the 0.25% DNJ and mixture groups indicated that the activity of trehalase was impaired, thus leading to the direct hydrolysis of trehalose [42]. The above change suggested turbulent energy and carbon mobilization mechanisms. Adenosine triphosphate (ATP) is a nucleoside triphosphate utilized as an energy supporter in the cells of all known organisms and it can transport chemical energy for metabolism [43]. In eukaryotic cells, the TCA reaction sequence is performed as the primary pathway for ATP production [44]. In our study, the decreased concentrations of the intermediates in TCA cycle, such as citrate, succinate, fumarate, and malate, indicated the depressive metabolic level of TCA cycle as well as the subsequent decline in the production of ATP and energy metabolism. Poor ATP production due to DNJ and mixture induced impaired TCA cycle affects not only cellular energy metabolism but also other pathways that require an adequate supply of ATP. The alteration in pathways namely, alanine, trehalose, citrate, succinate, fumarate, malate, glutamate and glutamine metabolism as revealed by our results could also be a consequence of disturbed mitochondrial metabolism, as metabolites involved in the energy metabolism are also important intermediates of many of the above mentioned pathways.

### Changes in the other metabolites

The concentrations of branched-chain amino acids (BCAAs), such as valine and leucine, were significantly changed in hemolymph of medicated Eri silkworms, but the consistent change pattern compared with age-matched controls were not found (Fig 4A and 4B). The difference suggests that BCAA metabolism pathway is different from that in normal stages. The loading plots of hemolymph in experimental Eri silkworms compared to the controls show the disturbed concentrations of several amino acids, such as glutamine, alanine, and valine (Fig 4B, 4D and 4F), indicating the altered degradation of proteins and glycoproteins [45]. The reduced level of tyrosine in hemolymph is only observed in the latex group (Fig 4P). Tyrosine is precursor of a variety of biologically important substances related to the tyrosine metabolism pathway. The reduced level of tyrosine indicated the attenuated pathway of tyrosine metabolism in Eri silkworms fed with latex [46]. Moreover, other metabolites related to fat metabolism, transamination, energy metabolism, and glycometabolism, such as glycine, o-phosphocholine, threonine, trigonelline, and histidine (Fig 4J–4M and 4Q), were turbulent in all the drug treatment groups. Nevertheless, these metabolites often showed the opposite tendency in the 0.25% DNJ group and the other two groups and the specific mechanism is unknown and requires further study.

## Conclusion

The present work was the first successful identification of metabolic changes in hemolymph from Eri silkworms, *S*. *cynthia ricini* by respectively feeding with H_2_O, 0.25% DNJ, latex and the mixture of 0.5% DNJ and latex through ^1^H-NMR-based metabonomic analysis. These biomarkers (leucine, valine, lactate, alanine, lysine, glutamine, succinate, citrate, malate, glycine, o-phosphocholine, threonine, trigonelline, trehalose, fumarate, tyrosine, and histidine) also proved that the 2-d feeding of DNJ or latex induced metabolic perturbations in hemolymph of Eri silkworms. Our results preliminarily indicate that DNJ is a secure substance as hypoglycemic medications due to its nontoxicity and bioactivity.

## Supporting Information

S1 Table Normalized intensity of metabolites in hemolymph obtained from newly molted fourth-instar larvae control and experimental Eri silkworms, *S. cynthia ricini*. (DOCX)

S1 Fig Representative 2D ^1^H-^1^H cosy spectra of hemolymph sample obtained from one control Eri silkworms. (JPG)

S2 Fig Representative 2D ^1^H-^1^H cosy spectra of hemolymph sample obtained from one 0.25% DNJ-fed Eri silkworms. (JPG)

S1 File This file contains all the NMR data of hemolymph in Eri Silkworm obtained in our work. (ZIP)
