# Supplementary material for: A 1H-NMR Based Study on Hemolymph Metabolomics in Eri Silkworm after Oral Administration of 1-Deoxynojirimycin
Source: PLoS One. 2015 Jul 6;10(7):e0131696. doi: 10.1371/journal.pone.0131696 (PMC4492494; doi:10.1371/journal.pone.0131696)
Supplement: S1 Table — (DOCX) [file pone.0131696.s001.docx]

| δ ^1^H (ppm) | Metabolites | H_2_O | 0.25% DNJ | Latex | The mixture of 0.5% DNJ and  latex |
| --- | --- | --- | --- | --- | --- |
| 0.95 | Leucine | 13.76±2.29 | 18.15±1.33**^**^** | 5.01±1.67**^**^** | 9.28±1.13**^**^** |
| 1.04 | Valine | 8.34±1.69 | 10.76±0.57**^**^** | 2.12±1.21**^**^** | 5.13±0.62**^**^** |
| 1.32 | Lactate | 1.60±0.47 | 2.5±0.77**^*^** | 3.17±3.20 | 2.62±1.14**^*^** |
| 1.48 | Alanine | 17.04±0.76 | 16.83±0.45 | 6.47±0.83**^**^** | 14.51±1.40**^**^** |
| 1.72 | Lysine | 10.01±0.60 | 12.04±0.55**^**^** | 8.53±1.68 | 8.09±0.44**^**^** |
| 2.13 | Glutamine | 29.72±1.68 | 27.75±0.69**^*^** | 13.44±2.79**^**^** | 16.26±0.90**^**^** |
| 2.40 | Succinate | 22.95±2.18 | 19.47±1.05**^**^** | 8.29±3.59**^**^** | 18.40±2.52**^**^** |
| 2.54 | Citrate | 21.14±1.12 | 14.51±1.03**^**^** | 4.11±0.74**^**^** | 9.46±0.82**^**^** |
| 2.66 | Malate | 8.04±0.92 | 7.56±0.42 | 2.21±1.97**^**^** | 5.32±0.47**^**^** |
| 3.56 | Glycine | 15.80±1.90 | 12.92±0.46**^**^** | 22.08±0.99**^**^** | 16.39±1.68 |
| 4.16 | O-phosphocholine | 9.61±0.85 | 7.05±0.42**^**^** | 24.29±4.61**^**^** | 12.81±1.09**^**^** |
| 4.25 | Threonine | 4.95±0.31 | 5.70±0.18**^**^** | 1.48±0.34**^**^** | 2.92±0.34**^**^** |
| 4.44 | Trigonelline | 0.67±0.06 | 0.47±0.05**^**^** | 1.42±0.25**^**^** | 0.55±0.04**^**^** |
| 5.19 | Trehalose | 15.78±1.21 | 18.61±0.64**^**^** | 14.95±3.71 | 24.00±2.06**^**^** |
| 6.52 | Fumarate | 4.23±0.34 | 4.22±0.13 | 1.41±0.82**^**^** | 2.94±0.23**^**^** |
| 7.19 | Tyrosine | 4.73±1.22 | 5.50±0.24 | 1.49±1.01**^**^** | 4.43±0.77 |
| 7.86 | Histidine | 10.82±0.24 | 9.74±0.30**^**^** | 13.88±1.30**^*^** | 10.38±0.52**^*^** |

Values are expressed as mean±SD

**^*^** and **^**^** respectively indicate the significant (*P*<0.05) and extremely significant differences (*P*<0.01) compared to the control group.
