# Supplementary figures and images for: A 1H-NMR Based Study on Hemolymph Metabolomics in Eri Silkworm after Oral Administration of 1-Deoxynojirimycin
Source: PLoS One. 2015 Jul 6;10(7):e0131696. doi: 10.1371/journal.pone.0131696 (PMC4492494; doi:10.1371/journal.pone.0131696)

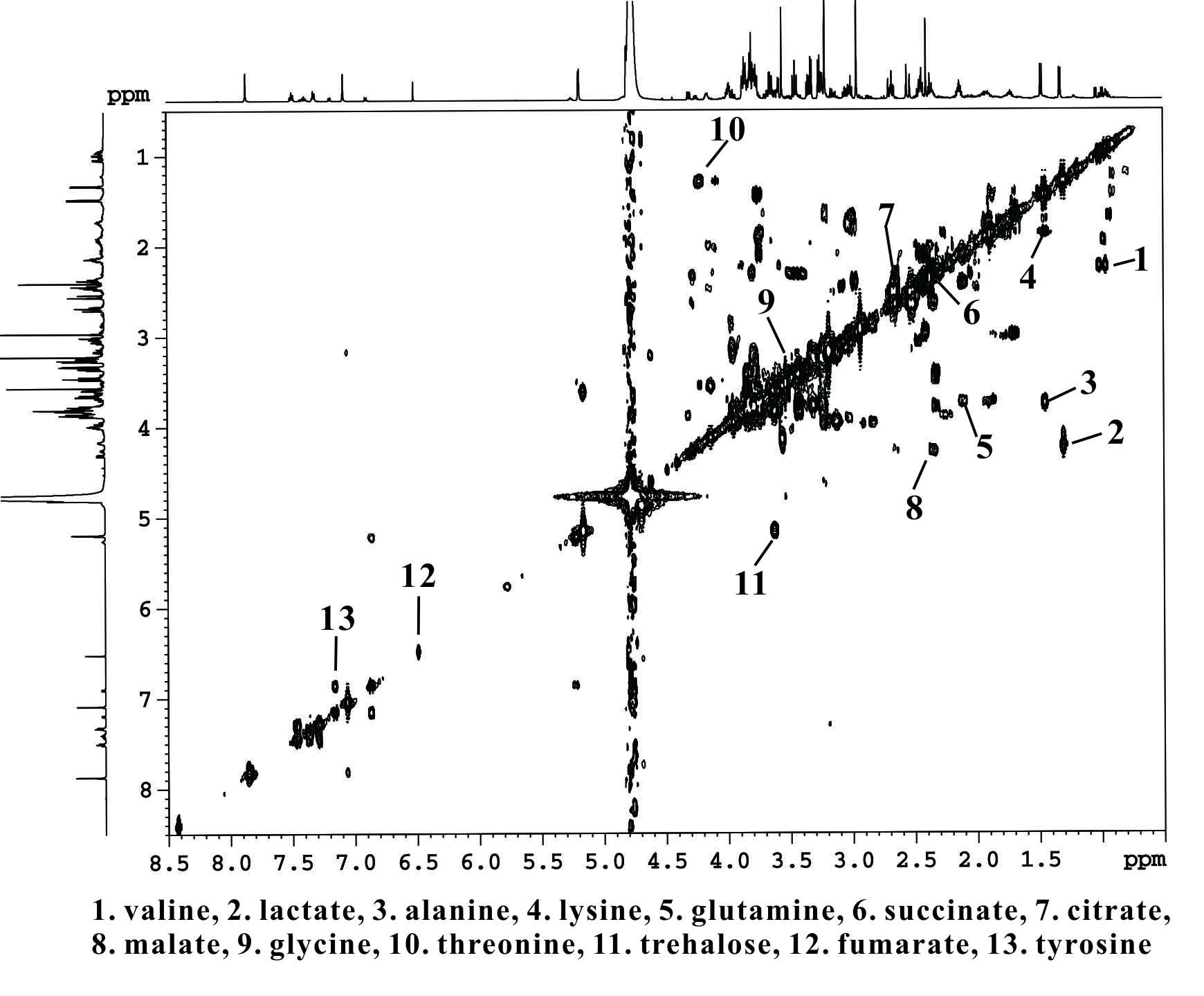

Supplement: S1 Fig — (JPG) [file pone.0131696.s002.jpg]

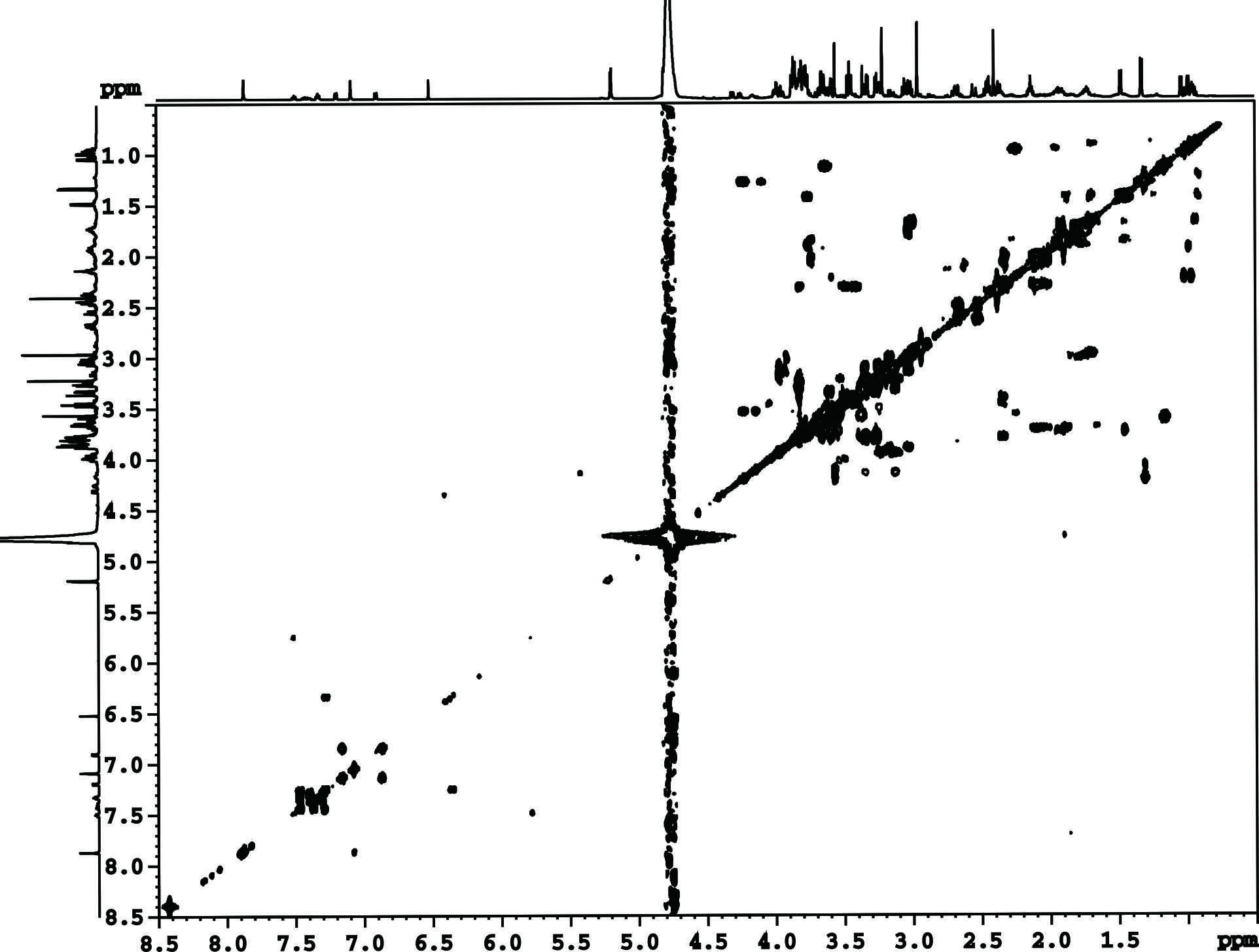

Supplement: S2 Fig — (JPG) [file pone.0131696.s003.jpg]
